# Fecal Fistula

**Published:** 1913-06

**Authors:** 


FECAL FISTULA. By John B. Deaver, M. D., Sc.D., LL.D., *The Therapeutic Gazette,* March 15, 1913. The causes of fecal fistula in the first instance may be illustrated by the following tabulation of 100 cases:

FINDINGS AT ORIGINAL OPERATION.

73 cases of appendiceal abscess.
9 cases of acute appendicitis with peritonitis.
4 cases of salpingitis.
3 cases of strangulated hernia.
1 case of peritonitis, unknown origin.
1 case of fibroid uterus and chronic appendix.
2 cases of perforated cecum, one typhoidal.
1 case of fecal fistula.
1 case of perforated ileum, typhoidal.
1 case of gall-stones.
2 cases of ovarian cyst.
1 case of umbilical abscess.
1 case of perinephric abscess.

Tuberculosis, actinomycosis, or carcinoma of the intestines may also be the starting point of a fecal fistula.

